# Insufficient Mechanical Loading Downregulates Piezo1 in Chondrocytes and Impairs Fracture Healing Through ApoE‐Induced Senescence

**DOI:** 10.1002/advs.202400502

**Published:** 2024-10-17

**Authors:** Siming Jia, Weijian Liu, Mo Zhang, Lijun Wang, Chuan Ren, Chen Feng, Tao Zhang, Hongzhi Lv, Zhiyong Hou, Weiguo Zou, Yingze Zhang, Wei Tong, Juan Wang, Wei Chen

**Affiliations:** ^1^ Department of Orthopaedic Surgery, NHC Key Laboratory of Intelligent Orthopaedic Equipment Hebei Medical University Third Hospital Shijiazhuang Hebei 050051 China; ^2^ Hebei Medical University Clinical Medicine Postdoctoral Station (Hebei Medical University Third Hospital) Shijiazhuang Hebei 050051 China; ^3^ Department of Orthopaedics Union Hospital Tongji Medical College Huazhong University of Science and Technology Wuhan Hubei 430022 China; ^4^ School of Pharmacy Key Laboratory of Innovative Drug Development and Evaluation Hebei Medical University Shijiazhuang 050017 China; ^5^ Hainan Institute of Regenerative Orthopedics and Sports Medicine, Hainan Academy of Medical Sciences and School of Basic Medicine Hainan Medical University Hainan 570000 China; ^6^ Key Laboratory of RNA Innovation, Science and Engineering, CAS Center for Excellence in Molecular Cell Science, Shanghai Institute of Biochemistry and Cell Biology, University of Chinese Academy of Sciences Chinese Academy of Sciences Shanghai 200031 China

**Keywords:** ApoE, endochondral ossification, fracture healing, mechanical loading, Piezo1

## Abstract

Insufficient mechanical loading impairs fracture healing; however, the underlying mechanisms remain unclear. Increasing evidence indicates that Piezo1 plays an important role in fracture healing, although the effect of Piezo1 on the endochondral ossification of chondrocytes has been overlooked. This study reports that mechanical unloading down‐regulates the expression of Piezo1 in chondrocytes and leads to fracture nonunion. Single‐cell sequencing of calluses revealed that specific deletion of Piezo1 in chondrocytes upregulated the expression of apolipoprotein E (ApoE) in hypertrophic chondrocytes, resulting in delayed cartilage‐to‐bone transition due to enhanced chondrocyte senescence. Based on these results, an injectable and thermosensitive hydrogel is developed, which released an ApoE antagonist in situ at the fracture site. This hydrogel effectively attenuated chondrocyte senescence and, thus, promoted cartilage‐to‐bone transition as well as the fracture healing process. Overall, this data provide a new perspective on the activity of chondrocytes in fracture healing and a new direction for the treatment of fracture nonunion caused by insufficient mechanical loading.

## Graphical abstract

**Graphic illustration of the findings of this study**. Insufficient mechanical loading downregulates Piezo1 expression in chondrocytes and impairs endochondral ossification during fracture healing. Specific deletion of Piezo1 in hypertrophic chondrocytes upregulates the expression of apolipoprotein E (ApoE), resulting in delayed cartilage‐to‐bone transition due to chondrocyte senescence.

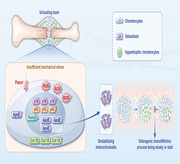

## 1 Introduction

Bone fractures are common injuries that affect people worldwide. In 2014, the population‐weighted incidence of bone fractures in China was 3.21 per 1000 people, and this incidence is increasing annually.^[^ ^1^ ^]^ Nonunion and delayed union are common complications of fracture healing, accounting for 5–10% of all fractures.^[^ ^2^ ^]^ In clinical practice, fracture healing can be impaired by insufficient mechanical loading during the postoperative fracture rehabilitation period, such as stress shielding caused by rigid fixation,^[^ ^3^ ^]^ or limited ambulation in the postoperative fracture rehabilitation period.^[^ ^4^ ^]^ However, the mechanism through which insufficient mechanical loading inhibits fracture healing remains unclear. A better understanding of the underlying mechanisms may reveal potential treatment strategies for fracture healing.

The two primary mechanisms of fracture healing are endochondral ossification and intramembranous ossification.^[^ ^2^, ^5^ ^]^ Endochondral ossification is the primary repair mode.^[^ ^6^ ^]^ In the early phase of fracture healing, stem cells such as periosteal skeletal stem/progenitor cells (PSCs) and mesenchymal stem cells (MSCs) migrate to the fracture site, where they differentiate into chondrocytes.^[^ ^5^, ^7^ ^]^ However, the mechanisms underlying this chondrogenic transdifferentiation remain unclear. The traditional theory of endochondral ossification proposes that chondrocytes undergo hypertrophic differentiation. Hypertrophic chondrocytes (HTCs) subsequently undergo apoptosis and are removed by osteoclasts during fracture healing.^[^ ^8^ ^]^ However, recent evidence has revealed that chondrocytes can transdifferentiate into osteoblasts.^[^ ^9^ ^]^ Long et al.^[^ ^10^ ^]^ used an HTC genetic reporter mouse model combined with single‐cell sequencing (scRNA‐seq) to explore the fate of HTCs during skeletal development. They found that in addition to apoptosis, HTCs differentiate in another direction by first dedifferentiating into immature chondrocytes and then re‐differentiating into osteoblasts and osteocytes.^[^ ^11^ ^]^ Zhou et al.^[^ ^11c^ ^]^ used Enhanced Green Fluorescent Protein (EGFP) and ROSA‐tdTomato to label hypertrophic chondrocytes and chondrocytes. The labeled cells were distributed throughout the trabecular surfaces and were later present in the endosteum and embedded within the bone matrix. Immunostaining for osteoblast markers (Col1) indicated that a proportion of the labeled chondrocytes transdifferentiated into osteocytes during growth and development. Hu et al.^[^ ^11b^ ^]^ performed lineage tracing experiments on Col2CreERT; Ai9 and Agc1CreERT; Ai9 mice. In both types of fluorescent Cre reporter mice, chondrocytes were labeled with red fluorescence on the growth plate and fracture calluses. The result of these lineage tracing experiments showed that chondrocytes could transdifferentiate into osteoblasts and osteocytes in the growth plate and during bone regeneration.

Piezo1 is a mechanosensing channel present in many cell types that plays an important role in bone formation.^[^ ^12^ ^]^ Piezo1 deletion in the developing skeleton results in bone malformations and spontaneous bone fractures, while the silencing of Piezo1 in adult mice causes osteoporosis.^[^ ^12^, ^13^ ^]^ In our previous study, we found that Piezo1 promotes the osteogenic, chondrogenic, and angiogenic capacity of PSCs by increasing the formation of the YAP/β‐catenin complex, which further promotes calluses formation and fracture healing.^[^ ^14^ ^]^ Piezo1 deletion in endothelial cells impairs Notch signaling and delays fracture repair.^[^ ^15^ ^]^ A previous study revealed that Piezo1 is required in chondrocytes for their transdifferentiation into bone‐forming osteoblasts. Col2a1Cre; Piezo1f/f mice exhibited decreased postnatal trabecular bone below the chondrogenic growth plate and developed multiple fractures of the rib bones at 7 days of age, which were located close to the growth plates.^[^ ^13^ ^]^ In contrast, OA pathology was markedly less pronounced in 60‐week‐old Col2a1Cre; Piezo1f/f mice than in littermate controls.^[^ ^16^ ^]^ Endochondral ossification is the process of bone formation through the replacement of the cartilage anlage, which is involved in skeletal growth, development, and bone fracture healing. However, it is unclear whether Piezo1 in chondrocytes plays the same role in fracture healing as in skeletal growth and development.

In the current study, we found that mechanical unloading downregulated the expression of Piezo1 in chondrocytes and led to fracture nonunion. Mice with a specific deletion of Piezo1 in chondrocytes (Piezo1Col2a1 mice) were used to explore the role of mechanical unloading in endochondral ossification. Single‐cell RNA sequencing (scRNA‐seq) of calluses revealed that the specific deletion of Piezo1 in chondrocytes upregulated the expression of Apolipoprotein E (ApoE) in HTCs, which resulted in chondrocyte senescence and delayed cartilage‐to‐bone transition. Based on the above results, we formulated an injectable and thermosensitive hydrogel for the sustained release of an ApoE antagonist in situ. Our results revealed that this hydrogel effectively ameliorated delayed fracture healing induced by mechanical unloading. Our findings highlight a new perspective on the role of chondrocytes in endochondral ossification, which might be targeted to treat fracture nonunions caused by insufficient mechanical loading.

## 2 Results

### 2.1 Insufficient Mechanical Loading Resulted in Impaired Fracture Healing by Downregulating the Expression of Piezo1 in Chondrocytes

**Figure 1:**
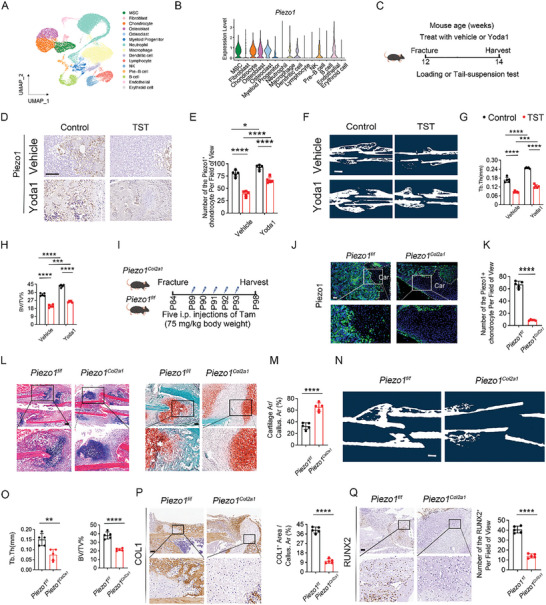
Insufficient mechanical loading impaired fracture healing by downregulating the expression of Piezo1 in chondrocytes. A) Single‐cell RNA sequencing (scRNA‐seq) analysis revealed 11 cell clusters isolated from calluses at 10 days and 20 days post fracture (dpf) as shown by UMAP plot. The name of each cell cluster is shown on the right. B) Violin plots of the expression levels of *Piezo1* genes in each cell cluster. C) Schematic showing the time‐course of the mouse femoral fracture experiment, under control or insufficient mechanical loading (tail‐suspension test; TST) conditions, treated with vehicle or the specific activator of Piezo1, Yoda1. D) Representative immunohistochemical (IHC) staining of Piezo1 expression in the calluses from the control and TST groups treated with vehicle or Yoda1 at 14 dpf. Scale bar: 50 µm. E) Quantitative analysis of Piezo1 expression in calluses from control and TST groups treated with vehicle or Yoda1 at 14 dpf. Two‐way ANOVA was performed. Data are represented as means ± SD; n = 5 mice per group, ^*^p < 0.05, ^***^ ^*^p < 0.0001. F) Representative µCT images of fractured femurs from control and TST groups treated with vehicle or Yoda1 at 14 dpf. Scale bar: 2 mm. G and H) Quantitative analysis of bone volume/total volume (BV/TV) and trabecular thickness (Tb. Th) of fractured femurs from control and TST groups treated with vehicle or Yoda1 at 14 dpf. Groups were compared by a two‐tailed Student's *t*‐test. Data are represented as means ± SD; n = 5 mice per group, ^**^ ^*^p < 0.001, ^***^ ^*^p < 0.0001. I) Schematic of tamoxifen injection in *Piezo1* ^Col2a^ and *Piezo1^f/f^* mice. *Piezo1* ^Col2a1^ and *Piezo1^f/f^* mice were treated with Tamoxifen at a dose of 75 mg k^−1^g by intraperitoneal injection subcutaneously for 5 consecutive days began at day 5 after modeling. J and K) Representative immunofluorescence (IF) staining and quantitative analysis of Piezo1 of calluses from the *Piezo1^Col2a1^* and *Piezo1^f/f^* groups at 14 dpf. Scale bar: 250 µm. Data are represented as means ± SD; n = 5 mice per group, Groups were compared by two‐tailed Student's *t*‐test, ^***^ ^*^p < 0.0001. L) Representative hematoxylin and eosin (HE) and safranin O/fast green (SO/FG) staining images of the calluses area of *Piezo1^Col2a1^* and *Piezo1^f/f^* groups. Scale bar: 250 µm. M) Quantitative analysis of the cartilage area as a percentage of the total calluses area. Data are represented as means ± SD; n = 5. Groups were compared by two‐tailed Student's *t*‐test. Data are represented as means ± SD; n = 5 mice per group, ^*^ ^*^p < 0.01. N) Representative µCT images of fractured femurs from *Piezo1^Col2a1^* and *Piezo1^f/f^* mice at 14 dpf. Scale bar: 2 mm. O) Quantitative analysis of BV/TV and Tb. Th of fractured femurs from *Piezo1^Col2a1^* and *Piezo1^f/f^* mice at 14 dpf. Groups were compared by a two‐tailed Student's *t*‐test. Data are represented as means ± SD; n = 5 mice per group, ^*^ ^*^p < 0.01. P) Representative IHC staining and quantitative analysis of the COL1 expression area as a percentage of the total calluses area from *Piezo1^Col2a1^* and *Piezo1^f/f^* mice at 14 dpf. Scale bar: 250 µm. Groups were compared by a two‐tailed Student's *t*‐test. Data are represented as means ± SD; n = 5 mice per group, ^***^ ^*^p < 0.0001. (Q) Representative IHC staining and quantitative analysis of RUNX2 expression in the calluses from *Piezo1^Col2a1^* and *Piezo1^f/f^* mice at 14 dpf. Scale bar: 250 µm. Two‐tailed Student's *t*‐test. Data are represented as means ± SD; n = 5 mice per group, ^*^ ^*^p < 0.01.

To investigate the expression of Piezo1 in each cell cluster during endochondral ossification, scRNA‐seq analysis was performed on calluses obtained 10 and 20 days post‐fracture (dpf). Based on the expression of marker genes, cell‐type annotation of the scRNA‐seq data was completed, showing that the calluses comprised MSCs, fibroblasts, chondrocytes, osteoblasts, osteoclasts, myeloid progenitor cells, neutrophils, macrophages, dendritic cells, lymphocytes, natural killer (NK) cells, pre‐B cells, B cells, endothelial cells, and erythroid cells (**Figure** **1**A; Figure S1A, Supporting Information). ScRNA‐seq data revealed that chondrocytes expressed higher levels of Piezo1 (Figure 1B), suggesting that Piezo1 in chondrocytes might play an important role in endochondral ossification. However, it was unclear whether insufficient mechanical loading‐induced fracture nonunion resulted from Piezo1 downregulation in chondrocytes. Immunohistochemical (IHC) staining showed downregulated Piezo1 expression in chondrocytes in the tail‐suspension test (TST) group, suggesting that reduced Piezo1 expression in chondrocytes is one of the underlying mechanisms of fracture nonunion. IHC staining also showed that Yoda1, a specific activator of Piezo1, reversed the decrease in Piezo1 expression caused by insufficient mechanical loading (Figure 1C,E). Hematoxylin and eosin (HE) and safranin O/fast green (SO/FG) staining revealed the arrest of the healing process in the TST group at 14 dpf. As shown by micro‐CT (µCT), the bone volume fraction (BV/TV) and trabecular thickness (Tb. Th) levels were significantly lower in the TST group than in the control group. However, fracture healing in the TST mice was improved by activating Piezo1 with Yoda1 (Figure 1F–H; Figure S1B,C, Supporting Information). Next, we generated Col2a1CreERT2; Piezo1f/f mice (referred to as Piezo1Col2a1), and Piezo1f/f mice to explore the in vivo role of Piezo1 in chondrocytes. Tamoxifen was administered to the Piezo1Col2a1 and Piezo1f/f groups to induce the specific deletion of Piezo1 in chondrocytes during fracture healing (Figure 1I). Knockout efficiency was verified by IF staining (Figure 1J,K). Hematoxylin and eosin (HE) and SO/FG staining revealed significantly larger cartilage areas in the calluses of Piezo1Col2a1 mice (Figure 1L,M). The results of µCT indicated that the Piezo1Col2a1 group developed significantly smaller mineralized calluses compared with the Piezo1f/f group. Quantitative analysis of µCT images indicated that Tb. The Th and BV/TV decreased significantly in the Piezo1Col2a1 group at 14 dpf (Figure 1N,O). IHC staining indicated that the expression of COL1 and RUNX2 in calluses areas was significantly decreased in the Piezo1Col2a1 group than in the Piezo1f/f group (Figure 1P,Q). Overall, these results confirm that Piezo1 expression in chondrocytes is a key modulator of mechanical loading‐induced fracture healing.

### 2.2 Specific Deletion of Piezo1 in Chondrocytes Inhibited Cartilage‐to‐Bone Transition as Well as Fracture Healing

**Figure 2:**
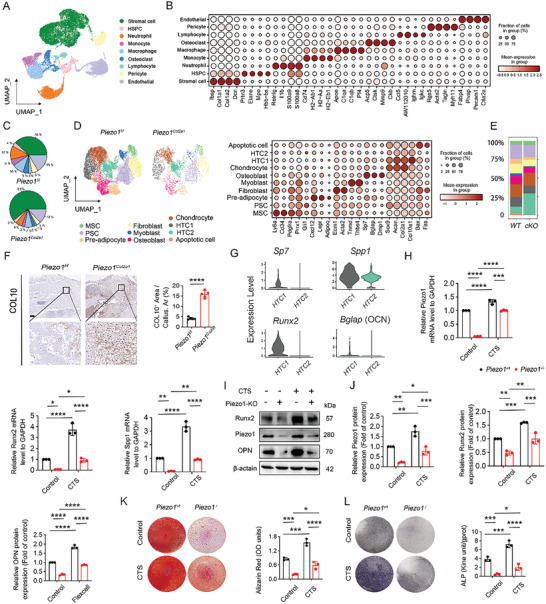
Specific deletion of Piezo1 in chondrocytes inhibited cartilage‐to‐bone transition and the fracture healing process. A) UMAP plot pooled from nine cell clusters isolated from calluses of *Piezo1^Col2a1^* and *Piezo1^f/f^* mice. The name of each cell cluster is shown on the right. B) Dot plot showing the specifically‐expressed genes of the major cell types. Red represents high expression; grey represents low expression. The size of the circle represents the percentage of cells that expressed the indicated genes. C) Pie chart showing the percentage of each cell type in *Piezo1^Col2a1^* and *Piezo1^f/f^* mice based on the UMAP distribution. D) UMAP plot (left image) and dot plot (right image) showing the re‐clustered cell subgroups and dot plot showing the specifically‐expressed genes of the major cell types isolated from the stromal cell subset. Red represents high expression; grey represents low expression. The size of the circle represents the percentage of cells that express the indicated genes. E) Stacked bar chart showing the percentages of each cluster isolated from the stromal cell subset. F) Representative IHC staining and quantitative analysis of the COL10‐positive area in the calluses from *Piezo1^Col2a1^* and *Piezo1^f/f^* mice at 14 dpf. Scale bar: 250 µm. Groups were compared using a two‐tailed Student's *t*‐test. Data are represented as means ± SD; n = 5 mice per group, ^***^ ^*^p < 0.0001. G) Violin plots showing the expression distribution of *Piezo1*, *Spp1* (OPN), *Runx2*, and *Bglap* (OCN) genes. H) RT‐PCR analysis of *Sp7*, *Runx2*, and *Spp1* gene expression in *Piezo1^wt^* and *Piezo1^−/−^* ATDC5 cells with or without cyclic tensile strain (CTS) treatment, n = 3 per group. Data are represented as means ± SD. Two‐way ANOVA was performed, ^*^p < 0.05, ^*^ ^*^p < 0.01, ^**^ ^*^p < 0.001, and ^***^ ^*^p < 0.0001. I and J) Western blotting analysis of Piezo1, RUNX2, and OPN protein expression in *Piezo1^wt^* and *Piezo1^−/‐^* ATDC5 cells with or without CTS treatment, n = 3 per group. Data are represented as means ± SD. Two‐way ANOVA was performed, ^*^p < 0.05, ^*^ ^*^p < 0.01, ^**^ ^*^p < 0.001, and ^***^ ^*^p < 0.0001. K and L) Alizarin Red S (ARS) staining, ALP staining, and quantitative analysis of *Piezo1^wt^* and *Piezo1^−/−^* ATDC5 cells cultured in osteogenic medium for 3 weeks with or without CTS; n = 3 per group. Data are represented as means ± SD. Two‐way ANOVA was performed, ^*^p < 0.05, ^*^ ^*^p < 0.01, ^**^ ^*^p < 0.001, and ^***^ ^*^p < 0.0001.

Based on the above results, scRNA‐seq was performed to investigate the transcriptional changes in calluses tissues between the *Piezo1^Col2a1^* group (n = 4) and the *Piezo1^f/f^* group (n = 6) at 14 dpf. A total of 27593 single cells were collected from calluses tissues for further analysis. Based on the expression of marker genes, we performed cell type annotation and identified nine cell clusters in our database, comprising stromal cells, pericytes, and endothelial cells, as well as immune cell clusters, including hematopoietic stem/progenitor cells (HSPCs), neutrophils, monocytes, macrophages, osteoclasts, and lymphocytes (**Figure** **2**A,B). Pie charts (Figure 2C) show the proportion of each cell subset, revealing that the proportion of immune and vascular cell subsets in the *Piezo1^Col2a1^* group was significantly decreased, indicating an abnormal immune microenvironment in the calluses.

We further analyzed the subclusters in the stromal cell population, which comprised the following cell subclusters: Ly6a(Sca‐1)^+^ Cd34^+^ MSCs, Gli1+ PSCs, Cxcl12+ Lepr^+^ Adipaq^+^ preadipocytes, Ecm1^+^ Acta2^+^ fibroblasts, Tnmd^+^ Thbs4^+^ myoblast, osteoblasts, chondrocytes, HTC cluster 1, HTC cluster 2, and apoptotic cells (Figure 2D). ScRNA‐seq analysis verified that the number of apoptotic cells, which are essential for the cartilage‐to‐bone transition, was reduced in the *Piezo1^Col2a1^* group. Notably, the proportion of HTCs drastically increased (especially HTC cluster 2) in the *Piezo1^Col2a1^* group and the number of osteoblasts decreased (Figure 2E). These results indicate that the deletion of Piezo1 in chondrocytes stagnates the process of HTC transdifferentiation into osteocytes. Col10 IHC staining was performed to confirm the *in‐silico* data. The results revealed that the Col10‐positive calluses area was significantly larger in the *Piezo1^Col2a1^* group than in the *Piezo1^f/f^* group (Figure 2F). Furthermore, scRNA‐seq analysis revealed that the expression of Piezo1 was significantly decreased in HTC cluster 2, indicating that HTC cluster 2 was a Piezo1 knockout‐induced subcluster (Figure S1D–F, Supporting Information). Accordingly, the expression of osteogenesis‐related genes, including *Sp7*, *Spp1* (encoding OPN), *Runx2*, and *Bglap* (encoding OCN), was significantly decreased in HTC Cluster 2 (Figure 2G). These results imply that Piezo1 knockout impaired the osteogenic capacity of HTCs.

Next, we introduced a stable Piezo1 deletion ATDC5 cell line (*Piezo1^−/−^*) to explore the function of Piezo1 in endochondral ossification in vitro. After 7 days of chondrogenic differentiation, the chondrogenic medium of *Piezo1^wt^* and *Piezo1^−/−^* ATDC5 cells was replaced with an osteogenic medium (Figure S2A, Supporting Information). The cells were subjected to cyclic tensile strain (CTS) on day 7 of chondrogenic induction by applying mechanical force. After osteogenic induction for an additional 3 days, Real‐time quantitative PCR (RT‐qPCR) and western blot assays revealed that CTS significantly upregulated *Runx2* and *Spp1* expression in *Piezo1^wt^* and *Piezo1^−/−^* ATDC5 cells, whereas Piezo1 deletion in chondrocytes significantly downregulated the expression of *Runx2* and *Spp1* compared with that in *Piezo1^wt^* ATDC5 cells (Figure 2H–J). Similarly, the results of Alizarin Red S (ARS) and alkaline phosphatase (ALP) staining also revealed that CTS increased the osteogenic differentiation capacity of *Piezo1^wt^* and *Piezo1^−/−^* ATDC5 cells, while deletion of Piezo1 impaired osteogenic differentiation (Figure 2K,L). Overall, these data suggest that the specific deletion of Piezo1 in chondrocytes results in delayed cartilage‐to‐bone transition.

### 2.3 Piezo1 Deletion Resulted in the Accumulation of ApoE in Chondrocytes

**Figure 3:**
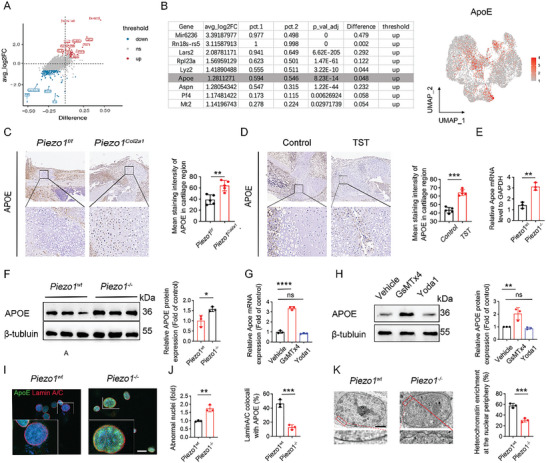
Piezo1 deletion resulted in the accumulation of ApoE in or around the nuclei of hypertrophic chondrocytes (HTCs), and caused disruption of heterochromatin. A) Differentially‐expressed genes (DEGs) in HTCs identified by scRNA‐seq in *Piezo1^Col2a1^* and *Piezo1^f/f^* groups. B) Top nine DEGs in the HTC cluster identified by scRNA‐seq analysis (left image). The cells were colored on the UMAP plot according to ApoE gene expression levels in the stromal cell population from *Piezo1^Col2a1^* and *Piezo1^f/f^* groups (right image). C) Representative IHC staining and quantitative analysis of the percentage of ApoE expression area from *Piezo1^Col2a1^* and *Piezo1^f/f^* groups at 14 dpf. Scale bar: 250 µm; n = 5 mice per group. Data are represented as means ± SD. Groups were compared using two‐tailed Student's t test, ^*^ ^*^p < 0.01. D) Representative IHC staining and quantitative analysis of the percentage of ApoE expression area from control and TST groups at 14 dpf; Scale bar: 250 µm; n = 5 mice per group. Data are represented as means ± SD. Groups were compared using two‐tailed Student's t test, ^**^ ^*^p < 0.001. E) RT‐PCR analysis of *Apoe* gene expression in *Piezo1^wt^* and *Piezo1^−/−^* ATDC5 cells; n = 3 per group. Data are represented as means ± SD. Two‐way ANOVA was performed, ^*^ ^*^p < 0.01. F) Western blotting analysis of ApoE gene expression in *Piezo1^wt^* and *Piezo1^−/−^* ATDC5 cells; n = 3 per group. Data are represented as means ± SD. Two‐way ANOVA was performed, ^*^p < 0.05. G) RT‐PCR analysis of Apoe gene expression in *Piezo1^wt^* ATDC5 cells treated with Yoda1 or the Piezo1 inhibitor, GsMTX4, n = 3 per group. Data are represented as means ± SD. Two‐way ANOVA was performed, ^***^ ^*^p < 0.0001, and ns: not significant. H) Western blotting analysis of ApoE gene expression in *Piezo1^wt^* ATDC5 cells treated with Yoda1 or GsMTX4, n = 3 per group. Data are represented as means ± SD. Two‐way ANOVA was performed, ^**^p < 0.01, and ns: not significant. I) Representative IF staining of ApoE and Lamin A/C in *Piezo1^wt^* and *Piezo1^−/‐^* ATDC5 cells. Scale bar: 20 µm. J) Quantitative analysis of abnormal nuclei (left) and percentage of Lamin A/C colocalized with ApoE (right) in *Piezo1^wt^* and *Piezo1^−/−^* ATDC5 cells. Data are represented as means ± SD, n = 3 per group. The two‐tailed Student's *t*‐test was performed, ^*^ ^*^p < 0.01, and ^**^ ^*^p < 0.05. Data are means ± SD of three independent experiments. K) TEM analysis of heterochromatin architecture at the nuclear periphery in *Piezo1^wt^* and *Piezo1^−/^* ^−^ATDC5 cells (left image). Quantitative analysis of the percentages of cells with heterochromatin enrichment at the nuclear periphery in *Piezo1^wt^* and *Piezo1^−/^* ^−^ATDC5 cells (right image). n = 3 for each group, data are represented as means ± SD. The two‐tailed Student's *t*‐test was performed, ^**^ ^*^p < 0.001. Data are means ± SD of three independent experiments.

To further explore the underlying mechanism of Piezo1 deletion‐reduced osteogenesis in HTCs, we examined the differences in gene expression between HTCs from the *Piezo1^Col2a1^* and *Piezo1^f/f^* groups and identified *ApoE* as a significantly upregulated gene in Piezo1 knockout HTCs (**Figure** **3**A,B). IHC staining revealed higher expression levels of ApoE in *Piezo1^Col2a1^* mice than in *Piezo1^f/f^* mice (Figure 3C), suggesting that ApoE is an important downstream effector of Piezo1 signaling in chondrocytes. Similarly, IHC staining verified that ApoE expression was significantly increased in HTCs in the calluses of the TST group (Figure 3D). We performed in vitro experiments to explore the relationship between Piezo1 and ApoE expression. RT‐qPCR and Western Blotting assays revealed that Piezo1 deletion significantly increased APOE expression in ATDC5 cells (Figure 3E,F). When chondrocytes were treated with GsMTX4, a Piezo1 inhibitor, the expression of ApoE was also significantly increased. However, ApoE expression did not differ significantly between the vehicle‐ and Yoda1‐treated ATDC5 groups (Figure 3G,H).

ApoE accumulation has been reported to result in the degradation of nuclear lamina proteins and disruption of heterochromatin in mesenchymal progenitor cells (MPCs). Therefore, we performed IF staining of ApoE and Lamin A/C to examine the effect of increased ApoE in chondrocytes. IF staining showed that the co‐localization of ApoE and the nuclear lamina area was significantly increased in Piezo1^−/−^ ATDC5 cells (Figure 3I,J). Similarly, transmission electron microscopy (TEM) results confirmed that the upregulated expression of ApoE in Piezo1^−/−^ ATDC5 cells resulted in disruption of nuclear architecture and heterochromatin organization (Figure 3K). These results collectively suggest that Piezo1 deletion may result in the accumulation of ApoE in chondrocytes and cause heterochromatin disruption.

### 2.4 Accumulation of ApoE Increased Senescence Level in Piezo1‐Deleted Chondrocytes

**Figure 4:**
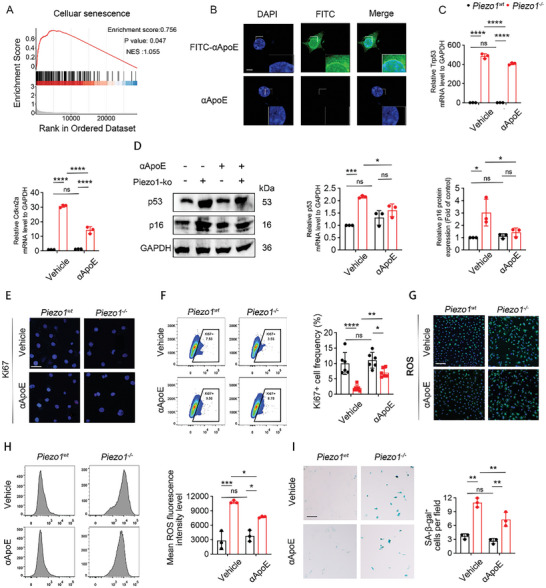
Activation of Piezo1‐ApoE signaling the increased senescence level of chondrocytes. A) Gene set enrichment analysis of the aging pathway of *Piezo1^Col2a1^* and *Piezo1^f/f^* groups. Top: The x‐axis shows genes ordered from high expression in the *Piezo1^Col2a1^* group (left end) to high expression in the *Piezo1^f/f^* group (right end). The y‐axis shows the running enrichment score (ES) along the ranked gene list. The negative ES near the left end indicates upregulation of the pathway in the *Piezo1^Col2a1^* group. Middle: black lines indicate the expression of genes of the aging pathway in the *Piezo1^Col2a1^* group relative to the ranked gene list. Bottom: metric for ranking genes based on the *Piezo1^Col2a1^* and *Piezo1^f/f^* groups. Nominal *p* value: 0.04692. B) Representative IF staining of FITC‐αApoE in ATDC5 cells. Scale bar: 10 µm. C) RT‐PCR analysis of *Cdkn2a and Trp53* gene expression in *Piezo1^wt^* and *Piezo1^−/−^* ATDC5 cells, n = 3 per group. Data are represented as means ± SD. Two‐way ANOVA was performed, ^***^ ^*^p < 0.0001, and ns: not significant. D) Western blotting analysis of p16 and p53 expression in *Piezo1^wt^* and *Piezo1^−/−^* ATDC5 cells, n = 3 per group. Data are represented as means ± SD. Two‐way ANOVA was performed, ^*^ *p* < 0.05, and ns: not significant. E) IF staining of Ki67 in *Piezo1^wt^* and *Piezo1^−/−^* ATDC5 cells. Scale bars, 50 µm. F) Representative flow cytometry image and quantitative analysis of the frequency of Ki67^+^ among *Piezo1^wt^* and *Piezo1^−/−^* ATDC5 cells, n = 3 for each group. Data are represented as means ± SD. Two‐way ANOVA was performed, ^*^p < 0.05, ^**^p < 0.01, ^**^ ^**^p < 0.0001, and ns: not significant. G) DCFH‐DA fluorescence analysis of *Piezo1^wt^* and *Piezo1^−/−^* ATDC5 cells. Scale bars, 100 µm. H) Representative flow cytometry image and quantitative analysis of mean fluorescence intensity of ROS in *Piezo1^wt^* and *Piezo1^−/−^* ATDC5 cells, n = 3 for each group. Data are represented as means ± SD. Two‐way ANOVA was performed, ^*^p < 0.05, ^**^ ^*^p < 0.001, and ns: not significant. I) Representative images of SA‐β‐Gal staining and quantitative analysis in *Piezo1^wt^* and *Piezo1^−/−^* ATDC5 cells, n = 3 for each group. Scale bars, 50 µm. Data are represented as means ± SD. Two‐way ANOVA was performed, ^*^ ^*^p < 0.01, and ns: not significant.

Considering that Piezo1 knockout induced heterochromatin disruption, bubble plot analysis was performed to explore the expression of senescence‐associated indicators in Piezo1‐specific deleted HTCs. Similarly, the expression of senescence‐associated indicators increased in HTC Cluster 2 (Figure S3A, Supporting Information). Gene set enrichment analysis (GSEA) of scRNA‐seq data showed upregulation of cellular senescence markers in the *Piezo1^Col2a1^* group than in the *Piezo1^f/f^* group (normalized enrichment score (NES) = 1.055; *p* = 0.047) (**Figure** **4**A). An ApoE antagonist (αApoE) was applied on day 7 of the induction process (Figure S3B, Supporting Information). Results evaluated using cell counting kit 8 (CCK8) revealed that 20 µM was the optimal drug concentration of αApoE for the treatment of *Piezo1^wt^* and *Piezo1^−/−^* ATDC5 cells in vitro, as it had no noticeable cytotoxic effects on *Piezo1^wt^* ATDC5 cells and increased the cell activity of *Piezo1^−/−^* ATDC5 cells (Figure S3C, Supporting Information). To observe the sites reached by the ApoE antagonist, αApoE was labeled with fluorescein isothiocyanate (FITC). The results of microscopic imaging showed that αApoE was able to penetrate the plasma membrane and reach the cytosol and nucleus of the cell (Figure 4B). In vitro, RT‐qPCR and Western Blotting assay also revealed increased expression of the senescence marker genes p53 and p16 in *Piezo1^−/−^* ATDC5 cells and the addition of αApoE (20 µM) alleviated the senescence phenotype after 10 days of treatment (Figure 4C,D). In addition, the proliferation marker Ki67 was examined by flow cytometry and IF staining. *Piezo1^−/−^* ATDC5 cells showed lower expression of Ki67, and αApoE increased their proliferative capacity (Figure 4E,F). Accordingly, *Piezo1^−/−^* ATDC5 cells displayed lower proliferative ability compared with *Piezo1^wt^* ATDC5 cells, and αApoE promoted proliferation ability in *Piezo1^−/−^* ATDC5 cells as evidenced by crystal violet staining (Figure S3D, Supporting Information). Next, we performed dichlorodihydrofluorescein diacetate (DCFH‐DA) fluorescence staining and flow cytometry to examine the ROS levels. The results showed increased ROS accumulation in *Piezo1^−/−^* ATDC5 cells while αApoE partially reversed this trend (Figure 4G,H). Accordingly, the proportion of β‐galactosidase‐positive cells was increased in *Piezo1^−/−^* ATDC5 cells and αApoE rescued the Piezo1 deletion‐induced senescence in *Piezo1^−/−^* ATDC5 cells (Figure 4I). Taken together, our results show that the accumulation of ApoE promotes cellular senescence in Piezo1‐deleted ATDC5 cells, suggesting that ApoE is a potential therapeutic target in fracture nonunion caused by mechanical unloading.

### 2.5 ApoE Antagonists Reverse the Osteogenic Inhibitory Effect of Piezo1 Deletion

**Figure 5:**
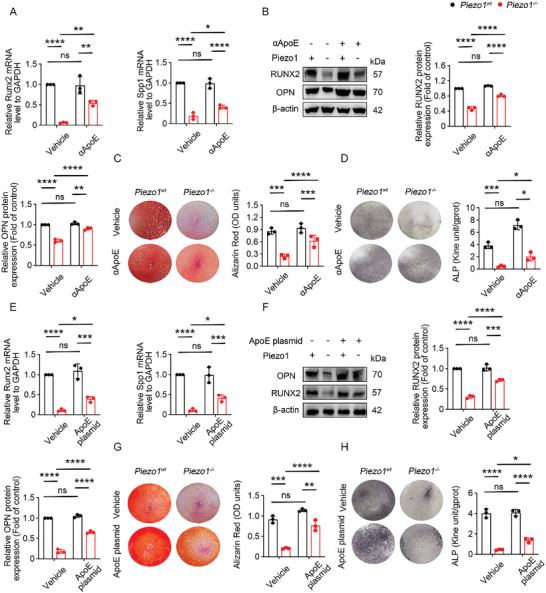
ApoE antagonists reversed the osteogenic inhibitory effect of Piezo1 deletion. A) RT‐PCR analysis of *Runx2* and *Spp1* gene expression in *Piezo1^wt^* and *Piezo1^−/−^* ATDC5 cells with or without αApoE treatment, n = 3 for each group. Data are represented as means ± SD. Two‐way ANOVA was performed, ^*^p < 0.05, ^*^ ^*^p < 0.01, ^***^ ^*^p < 0.0001, and ns: not significant. B) Western blotting analysis of RUNX2, and OPN protein expression in *Piezo1^wt^* and *Piezo1^−/‐^* ATDC5 cells with or without αApoE treatment, n = 3 per group. Data are represented as means ± SD. Two‐way ANOVA was performed, ^*^ ^*^p < 0.01, ^***^ ^*^p < 0.0001, and ns: not significant. C) ARS staining and quantitative analysis of *Piezo1^wt^* and *Piezo1^−/−^* ATDC5 cells cultured in osteogenic medium for 3 weeks with or without αApoE treatment, n = 3 per group. Data are represented as means ± SD. Two‐way ANOVA was performed ^**^ ^*^p < 0.001, ^***^ ^*^p < 0.0001, and ns: not significant. D) ALP staining and quantitative analysis of *Piezo1^wt^* and *Piezo1^−/−^* ATDC5 cells cultured in osteogenic medium for 3 weeks with or without αApoE treatment, n = 3 per group. Data are represented as means ± SD. Two‐way ANOVA was performed ^**^ ^*^p < 0.001, ^***^ ^*^p < 0.0001, and ns: not significant. E) RT‐PCR analysis of *Runx2 and Spp1* gene expression in *Piezo1^wt^* and *Piezo1^−/−^* ATDC5 cells with or without ApoE plasmid, n = 3 for each group. Data are represented as means ± SD. Two‐way ANOVA was performed, ^*^p < 0.05, ^*^ ^*^p < 0.01, ^***^ ^*^p < 0.0001, and ns: not significant. F) Western blotting analysis of RUNX2, and OPN gene expression in *Piezo1^wt^* and *Piezo1^−/‐^* ATDC5 cells with or without αApoE plasmid, n = 3 per group. Data are represented as means ± SD. Two‐way ANOVA was performed, ^*^ ^*^p < 0.01, ^***^ ^*^p < 0.0001, and ns: not significant. G) ARS staining, and quantitative analysis of *Piezo1^wt^* and *Piezo1^−/−^* ATDC5 cells cultured in osteogenic medium for 3 weeks with or without plasmid, n = 3 per group. Data are represented as means ± SD. Two‐way ANOVA was performed ^**^ ^*^p < 0.001, ^***^ ^*^p < 0.0001, and ns: not significant. H) ALP staining and quantitative analysis of *Piezo1^wt^* and *Piezo1^−/−^* ATDC5 cells cultured in osteogenic medium for 3 weeks with or without plasmid, n = 3 per group. Data are represented as means ± SD. Two‐way ANOVA was performed ^**^ ^*^p < 0.001, ^***^ ^*^p < 0.0001, and ns: not significant.

These findings suggest that insufficient mechanical loading results in senescence through the accumulation of ApoE in chondrocytes, which might impair the osteogenic transdifferentiation of HTCs.^[^ ^17^ ^]^ Therefore, we tested whether αApoE enhanced osteogenesis in *Piezo1^−/‐^* ATDC5 cells. After 10 days of induction, RT‐qPCR and Western Blotting results revealed that αApoE significantly increased the expression of Runx2 and Spp1 in *Piezo1^−/−^* ATDC5 cells (**Figure** **5**A,B). Accordingly, αApoE increased osteogenesis in *Piezo1^−/−^* ATDC5 cells as shown by ARS and ALP staining (Figure 5C,D). Similarly, to determine the functional role of ApoE in ATDC5 cells during osteogenic transdifferentiation of chondrocytes, ApoE knockout was performed in ATDC5 cells using the ApoE plasmid. The knockout efficiency of the ApoE plasmid was verified by RT‐qPCR and Western Blotting (Figure S3E,F, Supporting Information). RT‐qPCR and Western Blotting results revealed that ApoE plasmid significantly increased the expression of *Runx2* and *Spp1* in Piezo1^−/‐^ ATDC5 cells (Figure 5E,F). ARS and ALP staining also revealed that ApoE plasmid rescued the decreased osteogenic ability of Piezo1^−/−^ ATDC5 cells (Figure 5G,H). Thus, our results showed that ApoE antagonists increased the osteogenic capability of Piezo1^−/−^ ATDC5 cells.

### 2.6 Material Characterization of αApoE‐Loaded Hydrogels

**Figure 6:**
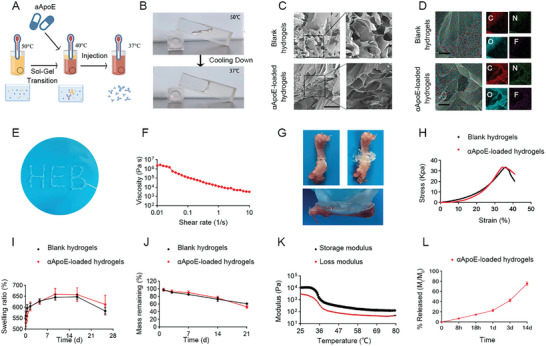
Materials characterization of αApoE‐loaded hydrogels. A) Schematic of αΑpoE‐loaded hydrogel fabrication. B) Optical images of the αApoE‐loaded hydrogels at different temperatures. C) Scanning electron microscopy (SEM) images of the internal structure of the blank hydrogels and αΑpoE‐loaded hydrogels, scale bar, 200 nm. D) Elemental mapping of carbon, nitrogen, oxide, and fluoride ions by EDS of the blank hydrogels and αΑpoE‐loaded hydrogels. Scale bar, 200 nm. E) Extrusion from a conventional medical syringe showing the injection performance of hydrogel scaffolds. F) Variation in viscosity with varying shear rate at the gelation temperature. G) Photo of αΑpoE‐loaded hydrogels adhering to the bone surface. H) Stress–strain curves for blank hydrogels and αΑpoE‐loaded hydrogels; n = 3 for each group, data are represented as means ± SD. The two‐tailed Student's *t*‐test were performed, and data are means ± SD of three independent experiments. I) Swelling ratio curves of blank hydrogels and αApoE‐loaded hydrogels; n = 3 for each group, data are represented as means ± SD. The two‐tailed Student's *t*‐test was performed, and data are means ± SD of three independent experiments. J) Mass remaining curves of blank hydrogels and αΑpoE‐loaded hydrogels; n = 3 for each group, data are represented as means ± SD. The two‐tailed Student's *t*‐test was performed, data are means ± SD of three independent experiments. K) Temperature‐dependent curves of storage modulus and loss modulus of αApoE‐loaded hydrogels, n = 3 for each group, data are represented as means ± SD. The two‐tailed Student's *t*‐test was performed, and data are means ± SD of three independent experiments. L) αApoE release over 14 days occurred at a rate proportional to the initial diffusion of drug loading in αΑpoE‐loaded hydrogels; 0.5 mL of αΑpoE‐loaded hydrogels (normalized) for each time point of release, n = 10 for each group, data are represented as means ± SD.

As the upregulation of ApoE levels upon Piezo1 deletion is likely a contributor to fracture non‐union under insufficient mechanical loading, we developed a type of thermoresponsive‐injectable hydrogel with the ability to provide sustained release of αApoE at a fracture site. A schematic of the thermoresponsive‐injectable hydrogel is shown in **Figure** **6**A. We injected the 50 °C hydrogel solution into a glass colorimetric dish. At this temperature, the hydrogel retained its high fluidity and spontaneously filled the bottom of the colorimetric dish. When the temperature was reduced from 50 to 37 °C, the hydrogel transitioned to a solid‐like state. This liquid–solid transition confirms that hydrogels are 3D networks formed by the crosslinking of hydrophilic polymer chains. This transition endows the hydrogels with the ability to maintain their shape and elasticity while absorbing water, thereby endowing them with high water absorption and retention capabilities (Figure 6B).

Notably, the in vivo injection temperature in our study was 40 °C, which had little effect on the surrounding tissues.^[^ ^18^ ^]^ Scanning electron microscopy (SEM) was used to examine the microstructures of the blank hydrogels and αApoE ‐loaded hydrogels (Figure 6C). After lyophilization, both hydrogels possessed a pore structure with internal connections, and there was little significant difference in microstructure between the blank hydrogels and αApoE‐loaded hydrogels. Energy dispersive spectroscopy (EDS) was also performed to explore the coexistence of αApoE and the distribution of αApoE within the hydrogel. The characteristic element peaks of the oxide and fluoride ions in the EDS spectra indicated that αApoE was successfully incorporated into hydrogels and distributed uniformly (Figure 6D).

The hydrogel was then injected into the molds using a syringe at 40 °C and solidified to form three hydrogel letters, thus validating the injectability and moldability characteristics of the hydrogel. The injectability of the hydrogel was demonstrated using a shear‐dependent viscosity test, and the results revealed prominent shear‐thinning properties (Figure 6E,F). This thermoresponsive‐injectable hydrogel showed improved interfacial adhesion between the hydrogel and bone interface, and the adhesion to the bone surface was around the fracture line, indicating its ability to bear the weight of the femur (Figure 6G). The mechanical properties of the hydrogels were analyzed in accordance with the stress–strain curves (Figure 6H). The two hydrogels exhibited similar mechanical properties, demonstrating that the addition of αApoE had little effect on their mechanical properties. In addition, there were no significant differences in swelling ratio or mass remaining between the blank hydrogel and the αApoE‐loaded hydrogel, indicating that αApoE did not affect the internal structure of the hydrogel (Figure 6I,J). Subsequently, a temperature sweep was performed using a heating process. Both the storage and loss moduli of the hydrogel gradually decreased during heating, whereas the storage modulus was always higher than the loss modulus, indicating that the disentanglement of the hydrogel network was dynamically slow (Figure 6K). Drug release curves were created by employing ninhydrin and showed that αApoE was continuously released at a nearly constant rate over 1 day, followed by a relatively slow‐release profile 1 day later. The cumulative release of αApoE exceeded 80% over 14 days, as shown in Figure 6L.

### 2.7 αApoE‐Loaded Hydrogels Promoted Delayed Fracture Healing Under Abnormal Mechanical Loading Conditions

**Figure 7:**
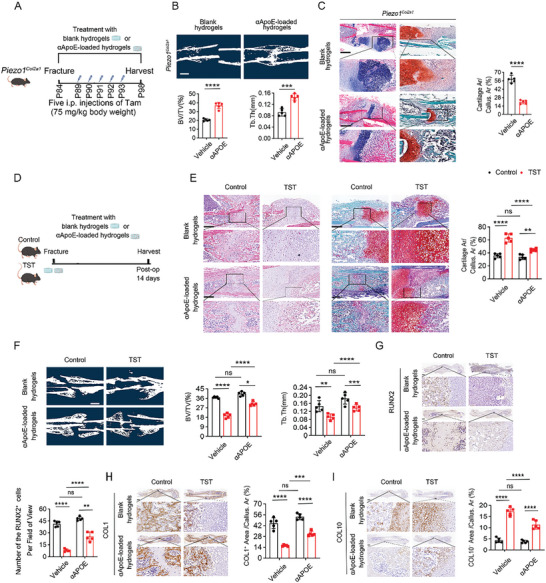
αApoE‐loaded hydrogels promoted delayed fracture healing under abnormal mechanical loading. A) Schematic showing the timeline of a fracture model study in *Piezo1^Col2a1^* mice treated with αΑpoE‐loaded hydrogel or blank hydrogel. After femoral fracture, *Piezo1^Col2a1^* mice were treated with αΑpoE‐loaded hydrogel or blank hydrogel. *Piezo1* ^Col2a1^ and *Piezo1^f/f^* mice were treated with Tamoxifen at a dose of 75 mg k^−1^g by intraperitoneal injection subcutaneously for 5 consecutive days began at day 5 after modeling. B) Representative µCT images and quantitative analysis of BV/TV and Tb. Th of fractured femurs in *Piezo1^Col2a1^* mice treated with αΑpoE‐loaded hydrogel or blank hydrogel at 14 dpf. Scale bar: 2 mm. Data are represented as means ± SD. Two‐tailed Student's *t*‐test was performed, ^*^ ^*^p < 0.01, ^***^ ^*^p < 0.0001, and ns: not significant. C) Representative HE and SO/FG staining images in the calluses area of *Piezo1^Col2a1^* mice treated with αApoE‐loaded hydrogel or blank hydrogel and quantitative analysis of the cartilage area as a percentage of total calluses area. Scale bar: 250 µm. Data are represented as means ± SD. Two‐tailed Student's *t*‐test was performed, ^*^p < 0.05, ^*^ ^*^p < 0.01, ^***^ ^*^p < 0.0001, and ns: not significant. D) Schematic of the timeline of a fracture model study in control and tail‐suspended mice treated with αΑpoE‐loaded hydrogel or blank hydrogel. After femoral fracture, male C57B6/J mice were allocated to a free movement or tail suspension group, and treated with αΑpoE‐loaded hydrogel or blank hydrogel. E) Representative HE and SO/FG staining images in the calluses area and quantitative analysis of the cartilage area as a percentage of the total calluses area. Scale bar: 250 µm. Data are represented as means ± SD. Two‐way ANOVA was performed, ^*^p < 0.05, ^*^ ^*^p < 0.01, ^***^ ^*^p < 0.0001, and ns: not significant. F) Representative µCT images and quantitative analysis of BV/TV and Tb. Th of the calluses of fractured femurs at 14 dpf. Scale bar: 2 mm. Data are represented as means ± SD. Two‐way ANOVA was performed, ^*^ ^*^p < 0.01, ^***^ ^*^p < 0.0001, and ns: not significant. G) Representative IHC staining and quantitative analysis of the percentage of RUNX2 expression in calluses area at 14 dpf. Scale bar: 500 µm. H) Representative IHC staining and quantitative analysis of COL1 expression in the calluses area at 14 dpf. Scale bar: 500 µm. Data are represented as means ± SD. Two‐way ANOVA was performed, ^*^p < 0.05, ^***^ ^*^p < 0.0001, and ns: not significant. I) Representative IHC staining and quantitative analysis of the COL10‐positive area in the calluses at 14 dpf. Scale bar: 500 µm. Data are represented as means ± SD. Two‐way ANOVA was performed, ns: not significant, and ^***^ ^*^p < 0.0001.

First, we administered the blank hydrogel and αApoE‐loaded hydrogel to *Piezo1^Col2a1^* mice. The results of µCT, HE and SO/FG staining showed that the αApoE‐loaded hydrogel played a certain role in promoting postoperative fracture healing upon Piezo1 knockout (**Figure** **7**A–C). Next, the αApoE‐loaded hydrogel was applied to the TST group to promote fracture healing under insufficient mechanical loading (Figure 7D). HE and SO/FG staining showed that the TST group developed an abnormal bone calluses and the transition of cartilage to bone occurred slowly. αApoE‐loaded hydrogels exerted osteogenesis‐promoting effects in the TST group (Figure 7E). The results of µCT analysis indicated that Tb. Th and BV/TV were both significantly decreased in the TST group than in the control group at 14 dpf, while αApoE‐loaded hydrogels enhanced the mineralization of calluses in the TST group (Figure 7F). IHC analysis showed abnormally increased COL10^+^ calluses areas in the TST group, and αApoE‐loaded hydrogels rescued this trend. Accordingly, the αApoE‐loaded hydrogels significantly promoted the expression of COL1 and RUNX2 in the calluses of the TST group (Figure 7G–I). In summary, these data confirm that fracture healing was delayed in mice under mechanical unloading conditions and that αApoE‐loaded hydrogels promoted fracture healing under insufficient mechanical loading.

## 3 Discussion

In clinical settings, insufficient mechanical loading such as prolonged bed rest after surgery or stress shielding caused by internal fixation impairs fracture healing. Our previous studies showed that the downregulated expression of Piezo1 was caused by insufficient mechanical loading and impaired chondrogenesis and osteogenesis of PSCs during endochondral ossification.^[^ ^19^ ^]^ Transdifferentiation of chondrocytes into osteoblasts is a primary cellular event in endochondral ossification.^[^ ^8b^ ^]^ However, whether insufficient mechanical loading downregulates Piezo1 expression in chondrocytes and impairs endochondral ossification remains unclear. In this study, we found that insufficient mechanical loading significantly downregulated the expression of Piezo1 in chondrocytes. In addition, our results confirmed that Piezo1 deletion in chondrocytes upregulated the expression of ApoE, resulting in chondrocyte senescence and delayed cartilage‐to‐bone transition. Based on the above results, we formulated an injectable and thermosensitive hydrogel capable of in situ release of an ApoE antagonist. This hydrogel effectively treated delayed fracture healing caused by insufficient mechanical loading. Collectively, these results provide a novel prospective therapeutic target for the treatment of insufficient mechanical loading‐induced delayed fracture healing.

Endochondral ossification is the primary method of fracture repair. However, the mechanisms of the cartilage‐to‐bone transition in calluses remain controversial. In classical theory, endochondral ossification begins with the hypertrophic differentiation of chondrocytes, followed by apoptosis and removal by osteoclasts.^[^ ^8a^ ^]^ Next, MSCs replace apoptotic chondrocytes and differentiate into osteoblasts.^[^ ^8d,e^ ^]^ This traditional theory highlights apoptotic outcomes in chondrocytes. In another study, Col10‐*CreER^T2^ ROSA26^fl/fl^* mice were used for lineage‐tracing experiments. Col10‐labelled cells substantially transdifferentiated into osteoblasts (Col1a1 marker) and osteocytes (sclerostin marker) during fracture healing. This study provided evidence that HTCs can dedifferentiate and redifferentiate into osteoblasts.^[^ ^20^ ^]^ Our study found that Piezo1 deletion in chondrocytes resulted in delayed cartilage‐to‐bone transition, verifying the osteogenic role of Piezo1 in chondrocytes.

As a lipoprotein complex, APOE plays important roles in lipid metabolism, storage, secretion, and delivery.^[^ ^21^ ^]^ Previous studies have demonstrated that ApoE accumulation drives cellular senescence,^[^ ^22^ ^]^ while ApoE deletion increases the resistance of MPCs to cellular senescence. Accumulation of ApoE activates the autophagy–lysosomal pathway, resulting in the degradation of nuclear lamina proteins and disruption of heterochromatin, ultimately leading to cellular senescence.^[^ ^17b^ ^]^ In our study, scRNA‐seq data verified that Piezo1 deletion in chondrocytes led to cellular senescence and inhibited the osteogenic ability of ATDC5 cells. ApoE antagonists reversed this trend in Piezo1 knock‐out ADTC5 cells. A previous study verified that increasing ApoE levels serve as an aging factor, impairing fracture healing by altering osteoblast metabolic processes.^[^ ^17a^ ^]^ Thus, we developed a thermoresponsive‐injectable hydrogel, which could be adhered to the broken ends of the fracture and provide sustained release of αApoE in situ. The results showed that the hydrogel effectively promoted fracture healing in an insufficient mechanical loading (TST) model. Thus, ApoE may be a possible therapeutic target for improving fracture healing.

This study has some limitations. First, due to ethical restrictions, we were unable to obtain human calluses samples. The majority of in vivo experimental data have been obtained from mice. Given the species‐specific differences between humans and mice, primate experiments should be conducted to validate the conclusions of the present study. Second, under the condition of Piezo1 deletion, the transdifferentiation process of HTCs to osteocytes stalled; however, further lineage tracing studies are required to verify our findings. Third, although our results revealed that *Piezo1* deletion inhibits fracture healing by upregulating ApoE expression, the nature of the intermolecular interactions between this mechanosensing channel and ApoE remains unknown and requires further investigation.

An insufficient mechanical loading impairs fracture healing. An optimized strategy that provides guidance for postoperative functional exercises and the application of suitable mechanical loading to the affected limb remains to be developed. In this study, we found that insufficient mechanical loading downregulated the expression of Piezo1 in chondrocytes, resulting in ApoE upregulation and delayed cartilage‐to‐bone transition. Our study proposes that the Piezo1–ApoE signaling pathway holds promise as a therapeutic target for fracture nonunion, especially when caused by insufficient mechanical loading.

## 4 Experimental Section

### Mice

All in vivo experiments were performed using 12‐week‐old male mice. Wild‐type C57BL/6J mice were obtained from Charles River (Beijing, China). *Col2CreER^T2^* mice were obtained from Cyagen Biosciences, Inc. (Guangzhou, China). *Piezo1^f/f^* mice were generously gifted by Prof. Weiguo Zou (Chinese Academy of Sciences, University of Chinese Academy of Sciences, Shanghai, China). To procure *Piezo1^Col2a1^* and *Piezo1^f/f^* mice, *Piezo1^f/f^* mice were mated with *Col2CreER^T2^* mice, and F1 littermates were mated with each other. PCR and western blot analyses were performed to determine the genotype of *Piezo1^Col2a1^* mice as described previously.^[^ ^23^ ^]^ The primers used for each genotype are listed in Table S1 (Supporting Information). All animal experiments were approved by the Ethics Committee of the Hebei Medical University (IACUC‐Hebmu‐P2222017).

### Femoral Fracture Model

Briefly, following the administration of isoflurane anesthesia (3% for induction and 1% for maintenance), the right thigh was shaved and disinfected. A transverse fracture of the mid‐femur was induced and inserted in a 23‐gauge needle into the medullary cavity for stabilization. At 14 dpf, mice were euthanized for sample collection. Harvested samples were preserved in 4% paraformaldehyde (PFA). Tamoxifen (75 mg per 1 kg body weight) was dissolved in corn oil and administered subcutaneously for 5 consecutive days to induce the deletion of Piezo1 in chondrocytes. According to a previous study,^[^ ^24^ ^]^ the chondrocytes of the calluses first appeared at approximately postoperative day 5 after modeling. Therefore, the first injection was performed in *Piezo1^Col2a1^* mice on day 5 after modeling (Figure 1I).

### Tail Suspension Test

To achieve unloading of the lower limbs, tail suspension experiments were performed immediately after fracture modeling. As previously described, fracture model mice were suspended at 30° to the tail to prevent the hind limbs from touching the ground.^[^ ^12a^ ^]^ The mice had free access to food and water. This posture was maintained for 14 days after surgery. Meanwhile, to rescue the low expression of Piezo1 in the TST group, Yoda1 (SML1558; Sigma, St. Louis, MO, USA) was injected daily for 5 consecutive days per week from modeling to sacrifice. The Yoda1 was dissolved in DMSO at 40 mM as a stock solution, then diluted in 5% ethanol, and injected intraperitoneally at 5 µmol kg^−1^ body weight.

### ScRNA‐seq and Analysis

Two sets of scRNA‐seq experiments were performed. The first scRNA‐seq experiment was performed using calluses harvested from C57BL/6J mice at 10 (n = 5) and 20 (n = 5) dpf. Calluses tissues from *Piezo1^Col2a1^* (n = 3) and *Piezo1^f/f^* (n = 3) mice were collected at 14 dpf and analyzed in another scRNA‐seq experiment. Enzyme digestion was used to dissociate all calluses samples into individual cell suspensions.^[^ ^25^ ^]^ cDNA amplification, chromium library construction, and library sequencing were performed (Lianchuan BioTechnology, Hangzhou, China). ScRNA‐seq data analysis was performed using the Seurat R package (version 3.1.4) as previously described.^[^ ^25^ ^]^ After screening, individual cells were grouped and visualized using a uniform manifold approximation and projection (UMAP) dimensionality reduction technique. Differentially expressed genes (DEGs) were determined by setting the adjusted *p*‐value threshold at 0.05 to define the DEGs. The enrichment score (ES) and normalized enrichment score (NES) were calculated for each gene set. The statistical significance of the NES was estimated by an empirical test using 1000 gene‐set permutations to obtain the nominal *p*‐value. All scRNA‐seq data were uploaded to the NCBI for Biotechnology Information Gene Expression Omnibus (GEO) database under the accession numbers GSE242712 and GSE266774.

### µCT Analysis

A SkyScan 1174 microcomputed tomography scanner (50 kV, 500 µA, 9 µm pixel^−1^) was utilized to scan the femoral samples. A 3D image of each sample was reconstructed using NRecon software (version 1.6, SkyScan; Microphotonics Inc., Allentown, PA, USA). CTVol v2.0 (SkyScan) was used to build a coronal plane image of the fracture calluses. CTAn (version 1.9, SkyScan) was used for quantitative analysis of the fracture calluses images. The parameters of µCT analysis were TV, BV, BV/TV, and Tb. Th.

### Histology and IHC/IF Staining

Femoral samples were dehydrated for 14 days using 10% ethylenediaminetetraacetic acid (EDTA, Solarbio Science & Technology, Beijing, China). A series of 5 µm serial paraffin‐embedded sections were cut and subjected to HE, SO/FG, IHC, and IF staining. Antigen retrieval was performed for IHC staining, followed by incubation of the sections with primary antibodies, including anti‐RUNX2 (1 1000, ab192256, Abcam, Cambridge, MA, USA), anti‐ApoE (1 100, PA5‐27088, Thermo Fisher Scientific, Waltham, MA, USA), anti‐Piezo1 (1 100, 15939‐1‐AP, Proteintech, Rosemont, IL, USA), anti‐collagen I (1 2000, ab270993, Abcam), and anti‐Collagen X (1 1000, ab182563, Abcam). IF staining was performed according to standard IF protocols.^[^ ^26^ ^]^ The following antibodies were used: anti‐Piezo1 (1:100, 15939‐1‐AP; Proteintech), anti‐ki67 (1:1000 dilution, ab92742; Abcam), anti‐lamin A/C (1:1000, sc‐376248; Santa Cruz Biotechnology, Santa Cruz, CA, USA), and anti‐ApoE (1:100, PA5‐27088; Thermo Fisher Scientific). A confocal microscope (A1; Nikon, Tokyo, Japan) and ImageJ software (NIH, Bethesda, MD, USA) were used for image capture and quantitative analyses.

### Cell Culture and Osteogenic Differentiation

*Piezo1^−/−^* ATDC5 cells were obtained from Cyagen Biosciences Technology. sgRNA plasmids were designed and constructed according to the gene sequence of Piezo1. The sgRNA used for *Piezo1^−/−^* ATDC5 cells was GTGCAGGCTGACAGGGCCCA‐GGG. The ATDC5 cell line was transfected with Cas9 and sgRNA plasmids via electroporation, and PCR was used to identify monoclonal cells with CRISPR/Cas9‐mediated Piezo1 deletion. Sanger sequencing was performed to confirm the homozygosity (Figure S4, Supporting Information). Table S2 (Supporting Information) lists the primers used for the PCR sequencing prism list of *Piezo1^−/−^* ATDC5 cells. A CCK8 assay (Abbkine, Redlands, CA, USA) was used to explore the effect of αApoE treatment on cell viability according to the manufacturer's instructions.

The osteogenic differentiation of *Piezo1^wt^* and *Piezo1^−/−^* ATDC5 cells was performed following the protocol reported by Hendrickx et al.^[^ ^13^ ^]^ In brief, after 7 days of chondrogenic differentiation, the chondrogenic medium of *Piezo1^wt^* and *Piezo1^−/−^* ATDC5 cells was replaced with an osteogenic medium. The chondrogenic medium was DMEM/F12 containing FCS (5%, Vivacell **Biotechnology,** Denzlingen, Germany), transferrin (10 µg mL^−1^, Beyotime), sodium selenite (30 nM, Sigma–Aldrich, St Louis, MO, USA), ascorbate‐2‐phosphate (0.2 mM, MedChemExpress (MCE), Monmouth Junction, NJ, USA), and insulin (10 µg mL^−1^, Beyotime). The osteogenic medium was α‐MEM containing FCS (10%, Vivacell **Biotechnology GmbH**) β‐glycerophosphate (10 mM, Sigma) ascorbate 2‐phosphate (0.2 mM, MCE), and recombinant BMP2 (100 ng mL^−1^, Sigma). To stimulate or block Piezo1, 50 mM DMSO was used to dissolve the Yoda1 (2 µM) or GsMTx4 (2.5 µM), which was added to the medium. The ALP activity of ATDC5 cells was measured using an Alkaline Phosphatase assay kit (Beyotime), and ALP staining was performed using a BCIP/NBT ALP color development kit (Beyotime) after 14 days of osteogenic induction. After 21 days of osteogenic induction, ARS staining (Alizarin Red S Solution, Solarbio Science & Technology) and quantitative analysis (ARed‐Q, ScienCell Research Laboratories, Carlsbad, CA, USA) were performed to visualize calcium deposition in *Piezo1^wt^* and *Piezo1^−/−^* ATDC5 cells. RNA lysates and protein samples were obtained 10 days after induction.

CTS experiments were performed on ATDC5 cells with or without Piezo1 in Collagen I‐coated BioFlex 6‐well culture plates (Flexcell International Corp., Burlington, NC, USA). After 7 days of chondrogenic induction, the cells were transferred to a Flexcell FX‐5000 Tension System (Flexcell International) and subjected to CTS (3%, 0.5 Hz). After 8 h of treatment, ATDC5 cells were collected for analysis.^[^ ^27^ ^]^

### ApoE Plasmid Mediated Deletion of the Apoe Gene

Deletion of ApoE was achieved using an ApoE plasmid. The ApoE plasmid was constructed using the CRISPR‐Cas9 ribonucleoprotein (RNP) complex (Haixing Bioscience) containing cassettes expressing hSpCas9 and chimeric guide RNA. Exons 1–2 of the ApoE gene were targeted with two sgRNA sequences, CTTCTGGGATTACCTGCGCT and GAAGACCCTAATGGACGCGA, selected from http://crispr.mit.edu. Plasmids containing the guide RNA sequence were transfected into cells using Lipo2000 (Invitrogen, Thermo Fisher Scientific), according to the manufacturer's instructions. This ApoE plasmid was able to maintain knockout efficiency during the osteogenic differentiation of *Piezo1^wt^* and *Piezo1^−/‐^* ATDC5 cells.

### RT‐qPCR Experiments

An RNeasy RNA extraction kit (Qiagen, Valencia, CA, USA) was used to extract total RNA from ATDC5 cells. The reverse‐transcription process was performed using GoScript Reverse Transcription Mix and Oligo dT primer (Promega Corporation, Madison, WI, USA). The mRNA expression levels were analyzed by RT‐qPCR using a one‐step RT ‐qPCR kit (Sangon Biotech, Shanghai, China). Table S3 (Supporting Information) lists the primers used for RT‐qPCR. The 2−ΔΔCt method was employed for data analysis.

### Western Blotting Experiments

A total protein extraction kit (Beyotime) was used to extract proteins from ATDC5 cells. The protein concentration was determined using a BCA Protein Assay Kit (Beijing Solarbio Science & Technology). Standard protocols were followed for Western Blotting. The antibodies used were anti‐Piezo1 antibody (1:1000, 15939‐1‐AP, Proteintech), anti‐RUNX2 (1:1000, ab192256, Abcam), anti‐ApoE (1:100, PA5‐27088, Thermo Fisher Scientific), anti‐p16 antibody (1:1000, ab241543, Abcam), anti‐p53 antibody (1:1000, ab241566, Abcam), anti‐ osteopontin (1:500, sc‐21742; Santa Cruz Biotechnology), β‐tubulin (1:10 000, ab7291, Abcam), β‐actin (1:5000, ab8227, Abcam), and secondary antibodies (1:5000, sc‐2357, Santa Cruz Biotechnology).

### αApoE Tracing Experiments

αApoE was purchased from MCE (HY‐P1050A). To observe the positional drug release behavior, green fluorescent protein (FITC) was used to label αApoE. FITC‐αApoE was provided by MCE. After ATDC5 cells were treated with FITC‐αApoE, the cells were washed trice with 1× phosphate‐buffered saline (PBS) and fixed in 4% PFA (methanol free) for 15 min, then washed twice with 1× PBS. Confocal microscopy (A1; Nikon, Tokyo, Japan) and ImageJ software were used for image capture and quantitative analysis.

### Clonal Expansion Assay

A crystal violet staining assay was used to evaluate cell proliferation. *Piezo1^w^* ^t^ and *Piezo1^−/−^* ATDC5 cells were cultured as described above. After fixing in 4% PFA, the cells were stained with 0.5% crystal violet (Sigma–Aldrich) for 20 min. Next, the cells were washed with PBS, crystal violet was extracted using 10% glacial acetic acid, and the OD_590_ was measured. To ensure reproducibility, a minimum of three growth assays were conducted with independent cell cultures used in each assay.

### SA‐β‐Gal Staining

After fixing in 4% PFA for 5 min, the cells were stained using the Senescence‐Associated β‐Galactosidase Stain Kit (Beijing Solarbio Science & Technology). Images were captured using an Olympus digital microscope camera and the number of SA‐β‐gal^+^ cells was determined using ImageJ.

### Transmission Electron Microscopy

TEM was performed as described by Zhao et al.^[^ ^17b^ ^]^ In summary, after digestion into single cells, *Piezo1^wt^* and *Piezo1^−/−^* ATDC5 cells were fixed with glutaraldehyde, dehydrated, embedded in resin, sectioned, and uranyl acetate and lead citrate staining were performed. A Hitachi TEM system (Spirit 80.0 kV) was used for image acquisition (Spirit 80.0 kV).

### Flow Cytometric Assay

*Piezo1^wt^* and *Piezo1^−/−^* cells were digested with pancreatin, fixed, and permeabilized using a Fixation and Permeabilization Kit (BD Biosciences, Franklin Lakes, NJ, USA). The cells were suspended in flow buffer and stained with anti‐ki67 antibody (1:100, 558616; BD Biosciences) for 30 min at 4 °C in the absence of light. Flow cytometry was performed using the Sony ID7000 flow cytometer (Sony Biotechnology, San Jose, CA, USA). FlowJo software (version 10.0, Treestar) was used to analyze the results.

### ROS Staining

ROS analysis was performed by staining the cells with DCFH‐DA (Beyotime Biotechnology, S0033S). The final concentration of DCFH‐DA was 50 µM. The ATDC5 cells were cultured as described above, then treated with DCFH‐DA and Hoechst 33342 for approximately 45–60 min at 37 °C in an atmosphere of 5% CO_2_. Subsequently, DCFH‐DA fluorescence was examined using a confocal microscope (Nikon A1) and a Sony ID7000 flow cytometer. The data were processed using ImageJ and FlowJo version 10.0 (Treestar).

### Hydrogel Preparation and Injection

Gellan gum (GG, 150 mg, Xieli Biotechnology Co. Ltd.) was dissolved in deionized water (5 mL) at a temperature of 90 °C. The solution was magnetically stirred until it became clear and completely dissolved. Next, αApoE was dissolved in DMSO, and then added to GG to reach a final concentration of 14 µM when the solution was cooled to 50 °C. For hydrogel treatment, 15 µL of αApoE‐loaded hydrogel at 40 °C was injected to cover the cortical bone surface around the fracture line after the anatomical reduction of the fracture. Subsequently, the αApoE‐loaded hydrogels were cooled to ambient temperature and solidified.

### Standard Curve of the Release of αApoE

Quantification of αApoE release in vitro was performed by ninhydrin staining of the peptide sequence of the drug (Leu‐Arg‐Val‐Arg‐Leu‐Ala‐Seu‐His‐Leu‐Arg‐Lys‐Leu‐Arg). Lys‐Arg‐Leu‐Leu generated a colorimetric reaction that varied in intensity depending on the concentration. The αApoE‐loaded hydrogel in a liquid‐state was placed into wells of a 24‐well plate (n = 10, 0.5 mL per well). After cooling the liquid‐state hydrogel to 37 °C, 2 mL of PBS was added to each well, and the plate was incubated at 37 °C for various durations (8, 18, or 24 h; 3, 7, or 14 days). Afterward, the liquid portion containing broken‐down hydrogel (200 µL) was collected. A standard curve was plotted and release testing was performed as described previously.^[^ ^28^ ^]^

### Structural and Compositional Analysis of the Hydrogels

Scanning electron microscopy (SEM; Quanta 450; FEI, Hillsboro, OR, USA) was used to analyze the porous structure and interconnectivity of the hydrogels. After snap‐freezing in liquid nitrogen and lyophilization, the hydrogels were sprayed with gold for subsequent SEM observation. Additionally, EDS mapping (20 kV) was performed to identify the specific elemental composition of the SEM images. The molecular formula of αApoE was C_99_H_182_F_3_N_37_O_21_. Of them, oxygen and fluorine were the specific elements that were not present in the hydrogels.

### Mechanical and Rheological Characterization

The samples were subjected to compression testing using a tensile‐testing machine (Dongguan Lixian Instrument Scientific Co., Ltd.). A load sensor was installed in the tensile‐testing machine at 10 N. The height and intensity changes of the hydrogels during the compression tests were recorded. A rheometer (MCR 302; Anton Paar, Graz, Austria) was used to perform rheological tests. To prevent water evaporation during the measurement, parallel plates (25 mm in diameter) were sealed using silicone oil, and the gap was set to 1.0 mm. Liquid hydrogel (1 mL) was placed onto parallel plates and allowed to react for 30 min. Except for the temperature sweeps, the experiments were conducted at a temperature of 37 °C, using a strain of 0.1% and a frequency of 1.0 Hz. To identify the linear viscoelastic regions, a strain‐scanning test was conducted in the strain range of 0.01–100%. Furthermore, the shear‐thinning properties were evaluated to explore the viscosity of the hydrogels at shear rates of 0.01–10 s^−1^. Temperature analysis was conducted within a temperature range of 25–80 °C, utilizing a heating rate of 2.0 °C min^−1^.

### Degradation and Swelling Behavior of Hydrogels

Three freeze‐dried hydrogels (Wd) were weighed and fully submerged in PBS. Samples were then removed and, after removing surface moisture, the samples were weighed at 0.083, 0.5, 2.5, 4.5, 9.5, 16.5, and 25.5 h (Ws). Swelling ratio (%) = (Ws‐Wd)/Wd × 100%.

The degradation of the blank hydrogel (n = 3) and αApoE‐loaded hydrogel (n = 3) was analyzed based on the weight loss of the PBS solution. The initial weight of the lyophilized hydrogel (W0) was measured and then the hydrogel was placed in a PBS solution at a temperature of 37 °C for durations of 1, 4, 7, 14, or 21 days. Subsequently, the hydrogel samples were removed, lyophilized, and weighed (W1). The degradation ratio was calculated as follows:

(1) $$\mathrm{Degradation}\;\mathrm{ratio}\left(\%\right)=\left[\left(W0-W1\right)/W0\right]\times 100\%$$

### Statistical Analysis

GraphPad Prism 8 software (GraphPad Software Inc., La Jolla, CA) was used to perform the data analysis and mapping in this study. According to the CLSI EP28‐A3 guidelines, outliers were eliminated using the Dixon range text.^[^ ^29^ ^]^ The Kolmogorov–Smirnov test was used to assess the normality of the data. The summary data of the experiments were presented as mean values ± the standard deviation (SD). At least five samples were tested in each group in vivo experiment and at least three per group were evaluated in the in vitro experiment. The two‐tailed Student's *t*‐test was used for two‐group comparisons and one‐way analysis of variance (ANOVA) with the Bonferroni post hoc test was used followed by multiple group analysis. Results were considered significant at α < 0.05, and two‐sided p values ≤0.05 were considered statistically significant.

## Conflict of Interest

The authors declare no conflict of interest.

## Author contributions

S.J., W.L., M.Z., and L.W. contributed equally to the study. W.C., J.W., WZ, and W.T. designed the study. S.J., W.L., M.Z., and L.W. performed the experiments and interpreted the data. C.R., C.F., T.Z., H.L., and Z.H. wrote the manuscript. W.Z. and Y.Z. reviewed the manuscript and made significant revisions. All the authors have read and approved the final version of the manuscript.

## Supporting information



Supporting Information

## Data Availability

The data that support the findings of this study are available from the corresponding author upon reasonable request.
