# Supplementary material for: Insufficient Mechanical Loading Downregulates Piezo1 in Chondrocytes and Impairs Fracture Healing Through ApoE‐Induced Senescence
Source: Adv Sci (Weinh). 2024 Oct 17;11(46):2400502. doi: 10.1002/advs.202400502 (PMC11633519; doi:10.1002/advs.202400502)
Supplement: Supplementary file 1 — Supporting Information [file ADVS-11-2400502-s001.docx]

**Supplementary figures**

**
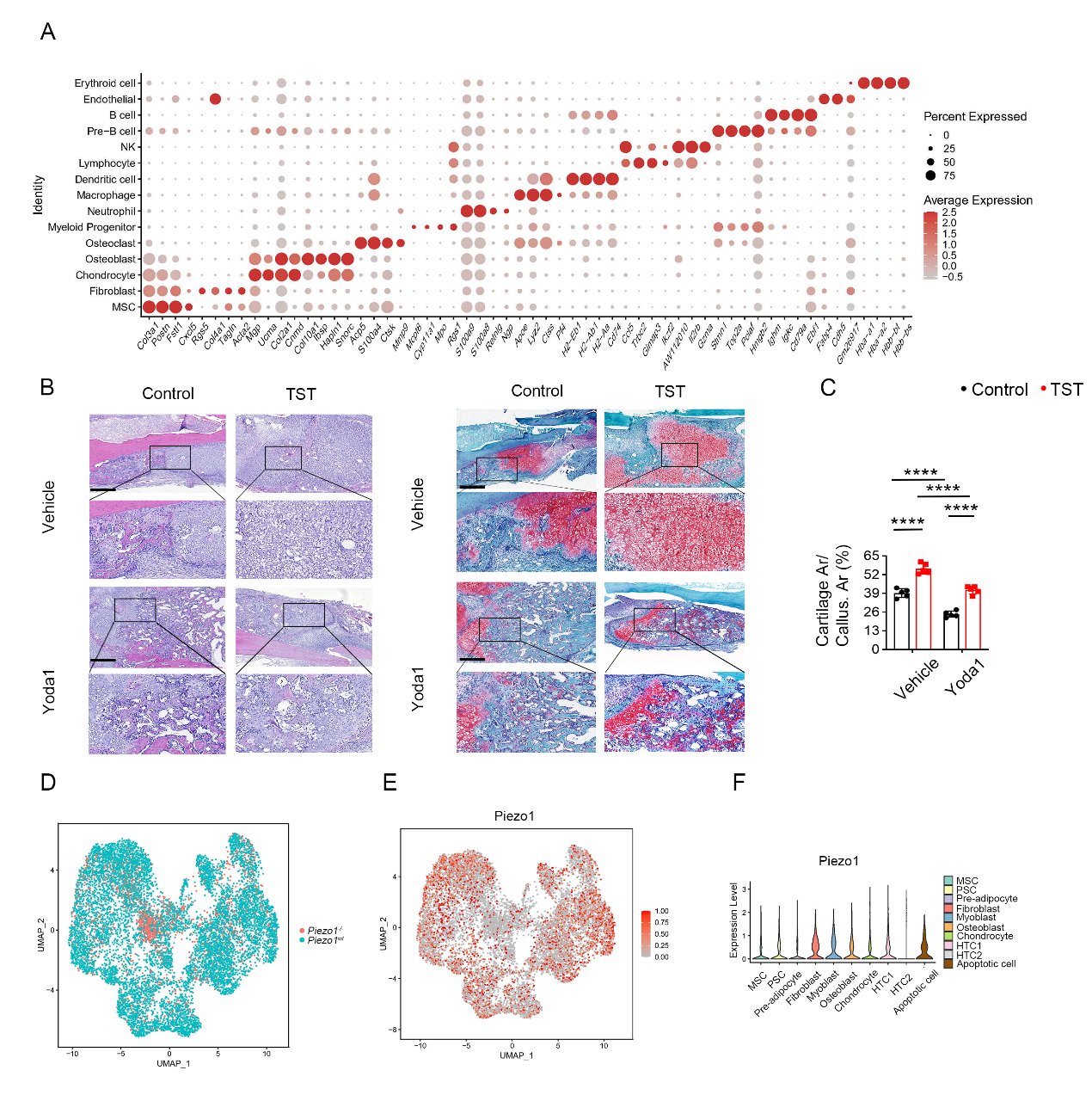
**

**Figure S1.** A) Dot plot showing the specifically-expressed genes of the major cell types. Red represents high expression; grey represents low expression. The size of the circle represents the percentage of cells that expressed the indicated genes. B) Representative images of HE and SO/FG staining of the calluses area of control and TST group mice treated with vehicle or Yoda1 at 14 dpf. Scale bar: 250 µm. C) Quantitative analysis of the cartilage area as a percentage of the total calluses area. Data are represented as means ± SD; n = 5. Two-way ANOVA was performed. Data are represented as means ± SD; n = 5 mice per group, ***p* < 0.01. D) UMAP plot showing the distribution of stromal cell across the *Piezo1^Col2a1^* and *Piezo1^f/f^* group. E) UMAP plot visualization of Piezo1 expression in the stromal cell subset. Red represents high expression; grey represents low expression. F) Violin plots showing the expression distribution of Piezo1 in MSCs, PSCs, pre-adipocytes, fibroblasts, myoblast, osteoblasts, chondrocytes, HTC cluster 1, HTC cluster 2, and apoptotic cells.

**
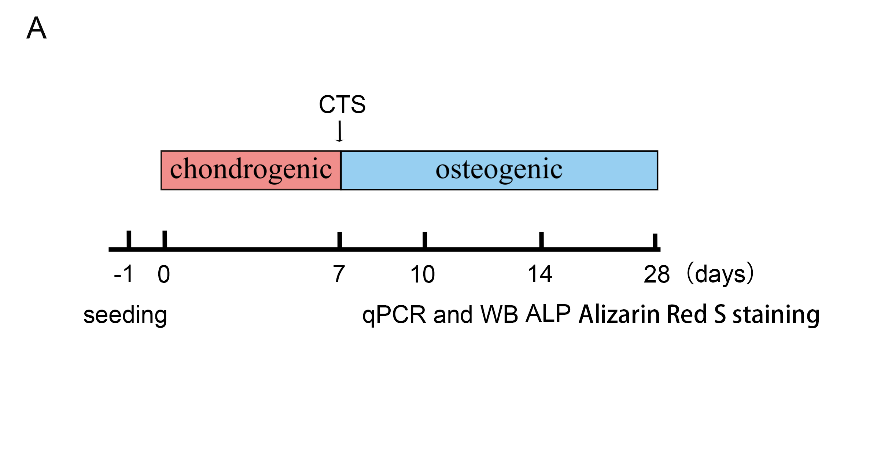
**

**Figure S2.** A) Setup of the *in vitro* endochondral ossification assay with differentiating ATDC5 cells treated with or without CTS (3%, 0.5 Hz).


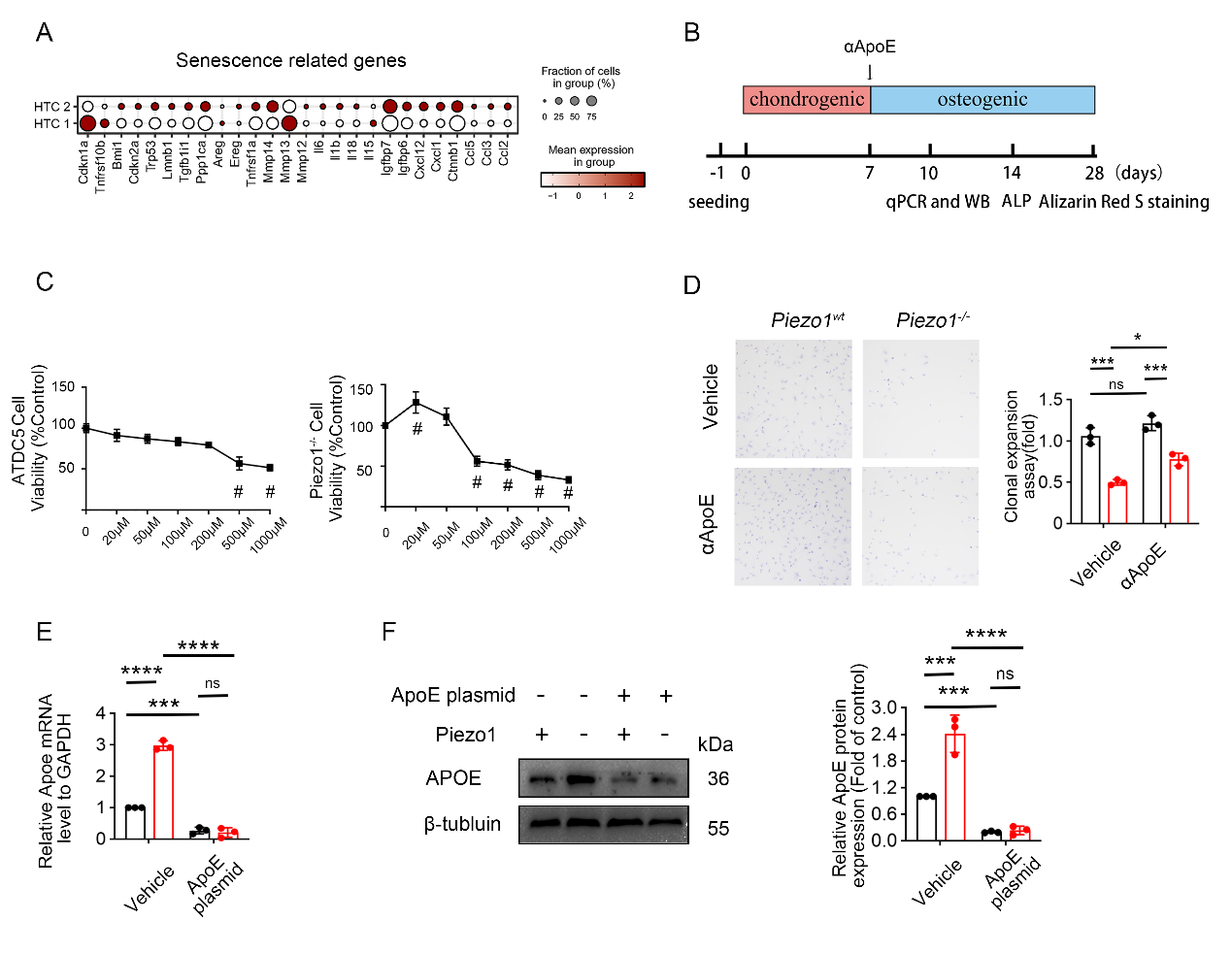


**Figure S3.** A) Expression of senescence-related genes in all clusters divided by condition and visualized in bubble plots. Intensity of the color (red) represents relative expression of a gene. Size of a bubble represents the percentage of cells in the cluster expressing that gene. B) Setup of the *in vitro* endochondral ossification assay with differentiating ATDC5 cells treated with or without αApoE (20 µM). C) CCK8 was used to determine the effect of αApoE in *Piezo1^wt^ and Piezo1^-/-^* ATDC5 cells *ex vivo*. For Piezo1^wt^ cells, higher doses of αApoE (more than 200 µM) inhibited their proliferation. A low dose of αApoE (20 µM) increased the Piezo1^-/-^ ATDC5 cell activity, while higher doses (more than 100 µM) inhibited cell activity in Piezo1^-/-^ ATDC5 cells. Therefore, we chose 20 µM as the concentration used for subsequent *in vitro* experiments. D) Clonal expansion assay was performed by crystal violet staining and results were quantified by measuring the absorbance of crystal violet at 595 nm. E) RT-PCR analysis of Apoe gene expression in *Piezo1^wt^* and *Piezo1^-/-^*ATDC5 cells with or without ApoE plasmid; n = 3 for each group. Data are represented as means ± SD. Two-way ANOVA was performed, **p* < 0.05, ***p* < 0.01, *****p* < 0.0001, and ns: not significant. F) Western blotting analysis of ApoE gene expression in *Piezo1^wt^* and *Piezo1^-/-^* ATDC5 cells with or without αApoE plasmid, n = 3 per group. Data are represented as means ± SD. Two-way ANOVA was performed, ***p* < 0.01, *****p* < 0.0001, and ns: not significant.


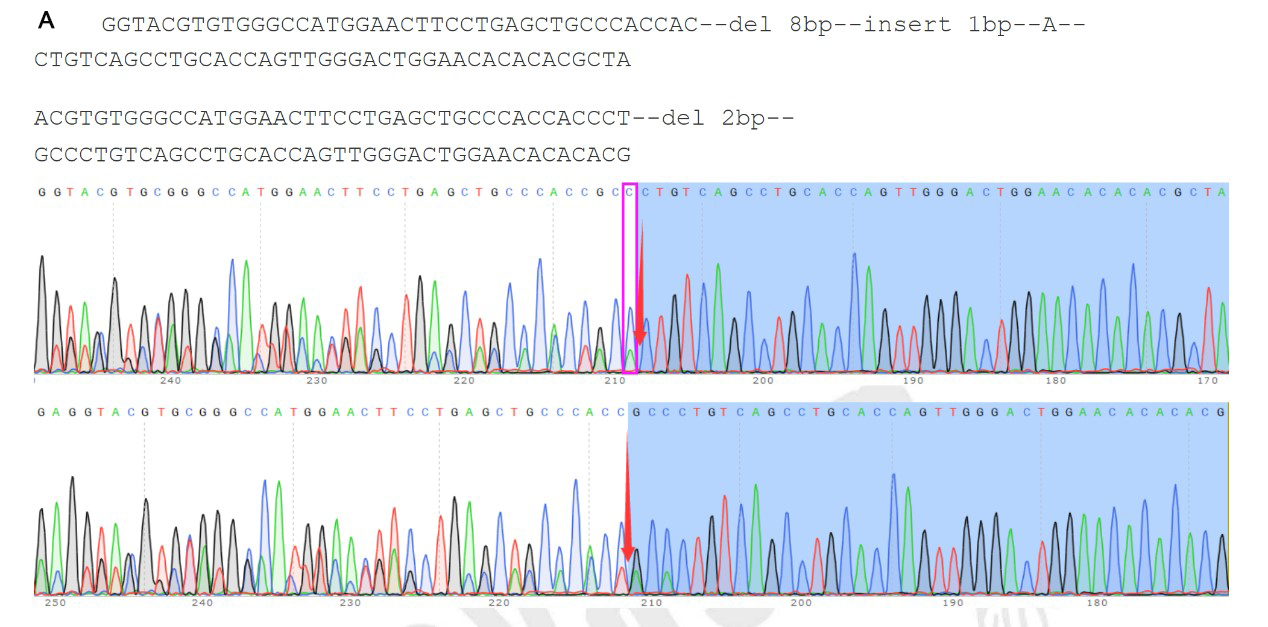


**Figure S4.** A) The Sanger sequencing results of *Piezo1^-/-^* ATDC5 cells.

**Table S1. Genotyping Conditions and PCR Primers**

| Primer Name | Forward (5'-3') | Reverse (5'-3') |
| --- | --- | --- |
| *Piezo1^f/f^* | CCAGTGATTCCTCATGGAATGTGG | CTTAAGCCCATCTCACAGCTGAAGG |
| *Col2a1CreER^T2^* | GGCTCTACTTCATCGCATTCCTTG | CGCAAACAAGTCTCACAAAGGAG |

**Table S2. Sequences of primers used for PCR sequencing**

| Forward primer (5′-3′) | Reverse primers (5′-3′) |
| --- | --- |
| AGATGTCACCGCTACATGGC | CATACTCGGTCACAACGCCT |
| ACATACTCGGTCACAACGCC |  |

**Table S3. Sequences of primers used for RT-qPCR**

| Gene | Forward primer (5′-3′) | Reverse primers (5′-3′) |
| --- | --- | --- |
| *Piezo1* | GAATGTGATTGGGCAGCGTATGAAC | GAACAGCGTGAGGAACAGACAGTAG |
| *SPP1* | TTCAATGGGCAGTTTTGAGC | ACTTACAAAACCGCCAAGC |
| *Cdkn2a* | CGCAGGTTCTTGGTCACTGT | TGTTCACGAAAGCCAGAGCG |
| *Trp53* | CCCCTGTCATCTTTTGTCCCT | AGCTGGCAGAATAGCTTATTGAG |
| *Runx2* | CCGCACGACAACCGCACCAT | CGCTCCGGCCCACAAATCTC |
| *Apoe* | CTCGGCAAGGGGAGGTAAAC | CTCGGCAAGGGGAGGTAAAC |
| *GAPDH* | GGGGAGCCAAAAGGGTCATCATCT | GAGGGGCCATCCACAGTCTTCT |
